# Influence of Intermediate Annealing Treatment on the Kinetics of Bainitic Transformation in X37CrMoV5-1 Steel

**DOI:** 10.3390/ma14164411

**Published:** 2021-08-06

**Authors:** Grzegorz Łukaszewicz, Krzysztof Wasiak, Emilia K. Skołek, Ryszard Diduszko, Wiesław A. Świątnicki

**Affiliations:** 1 Faculty of Materials Science and Engineering, Warsaw University of Technology, Wołoska 141 Street, 02-507 Warsaw, Poland; krzysztof.wasiak@pw.edu.pl (K.W.); emilia.skolek@pw.edu.pl (E.K.S.); wieslaw.swiatnicki@pw.edu.pl (W.A.Ś.); 2 Łukasiewicz Research Network, Tele & Radio Research Institute, Ratuszowa 11 Street, 03-450 Warsaw, Poland; ryszard.diduszko@itr.org.pl

**Keywords:** bainitic transformation, the kinetics of phase transformations, intermediate annealing treatment, tool steel, carbides

## Abstract

Intermediate annealing treatment (IAT) is a new process that accelerates the bainitic transformation in steels. This stimulation is crucial, especially in the prolonged production of nanobainitic steels. Among other recognised methods, it seems to be an effective and economical process. However, there are very few research works in this area. The objective of this study was to collate microstructural changes caused by IAT with differences in the kinetics of the subsequent bainitic transformation in the X37CrMoV5-1 tool steel. Differential dilatometry, LM and SEM microscopic observations, EDS and XRD analysis, and computer simulations were used to investigate the effect of IAT on the kinetics of bainitic transformation. The study has revealed that introducing an additional isothermal heating stage immediately after austenitising significantly affects the kinetics of bainitic transformation—it can accelerate or suppress it. The type and strength of the effect depends on the concentration, distribution, and morphology of the precipitations that occurred during IAT.

## 1. Introduction

Over the past few years, the formation of nanobainitic structures in steels has been the subject of many research works carried out in scientific centres worldwide [1,2,3,4]. This kind of structure can be obtained through isothermal quenching at a temperature slightly above the martensitic transformation start temperature M_s_. Nanobainite consists of bainitic ferrite plates of nanometric thickness separated by thin films of retained austenite. This microstructure ensures the favourable combination of strength, fracture toughness, and ductility comparable to much more expensive maraging steels [1,5]. For example, the tensile strength of nanobainitic steels can reach 2.3 GPa, hardnesses of 600–670 HV can be achieved, and the elongation is between 5% and 30% [1,6,7]. Moreover, nanobainitic steels have good service properties such as wear resistance [8,9] and fatigue strength [8,10]. These favourable properties are the reason for the ever-growing interest in nanobainitic steels for industrial applications.

Such beneficial mechanical properties are obtained by low temperature austempering. This heat treatment results in a nanometric two-phase microstructure made of bainitic ferrite and retained austenite. To achieve such a significant refinement of the microstructure, it is necessary to conduct austempering processes at relatively low temperatures in the range of 200–300 °C [4,11,12]. However, as the austempering temperature decreases, the kinetics of bainitic transformation slows down, which is unfavourable in industrial heat treatment processes. The long austempering time significantly reduces the efficiency of production and, up to now, is the main reason for the limited use of nanobainitic steels in industry [13].

To extend the use of the nanobainitic steels into industrial practice, many scientific groups have researched methods that reduce the time of the heat treatment by accelerating the bainitic transformation. Several approaches have been proposed in the literature to solve this problem. One solution is to add alloying elements such as aluminium and cobalt in up to 2 wt %. This leads to an acceleration of the bainitic transformation due to the increase in the free energy G of the austenite transformation into ferrite [1,14]. The increase in the bainitic transformation rate can also be achieved by reducing the content of manganese or by introducing a certain amount of nitrogen to form nitrides that constitute the additional nucleation sites of bainitic ferrite [15]. The second solution consists of introducing an extra step in the heat treatment process which allows the formation of a small amount of martensite before the bainitic transformation [16]. The martensite plates/laths serve as the auxiliary sites for the heterogeneous nucleation of bainitic ferrite. The third solution is the refinement of primary austenite grains (PAGs), which leads to an increase in the grain boundary density. The austenite grain boundaries can act as nucleation sites for bainitic ferrite. Therefore, an increase in their density shortens the transformation incubation time [14,17]. Finally, an acceleration of bainitic transformation is also observed when the ausforming process precedes the austempering. In this case, the kinetics of bainitic transformation depends on the ausforming temperature and the degree of deformation [18,19]. There are also other factors that can affect the bainitic transformation rate, such as additional stresses below the yield strength, or the application of a magnetic field during bainitic transformation [20,21].

The fact that a multitude of ways to stimulate bainitic transformation have been developed confirms the essence of the problem. However, the proposed methods have not found wide application in industry so far. This is mainly due to the additional costs and because they complicate the production process. The introduction of an extra stage that leads to a partial martensitic transformation requires additional equipment and proper cooling media. Different advanced devices are also required when applying a magnetic field or introducing stresses before the austempering.

A relatively new way to accelerate bainitic transformation is to use an intermediate annealing treatment (IAT) between the austenitising and bainitising steps. As demonstrated by Ravi et al., the use of IAT allows the hastening of the bainitic transformation in low- and high-carbon steels [22,23]. However, their research, which is among the first, was only the beginning of the surveys devoted to IAT. The influence of IAT in medium-carbon alloy steels, especially those containing carbide forming elements such as chromium, is still unknown. This is an important subject, especially since for this type of steel a nanobainite structure is effectively achieved [24,25].

In this work, the impact of the IAT parameters on the kinetics of bainitic transformation is investigated. Moreover, the microstructural changes induced by IAT are analysed. Finally, based on the obtained results, an explanation for the observed phenomena is proposed.

## 2. Materials and Methods

The investigated material was the hot-work tool steel EN X37CrMoV5-1. In the initial state, the steel had a spheroidite microstructure. The share of the main elements (wt %) except iron is presented in Table 1. The selected steel grade exhibits many advantages that are favorable from the point of view of this research. Steel EN X37CrMoV5-1 shows excellent hardenability. Moreover, the temperature–time ranges of the pearlitic and bainite transformations are separated by the bay of austenite stability. This allows IAT variants to be carried out over a wide range of temperatures and times. Furthermore, it is possible to obtain nanobainite in this steel [25].

**Figure 1:**
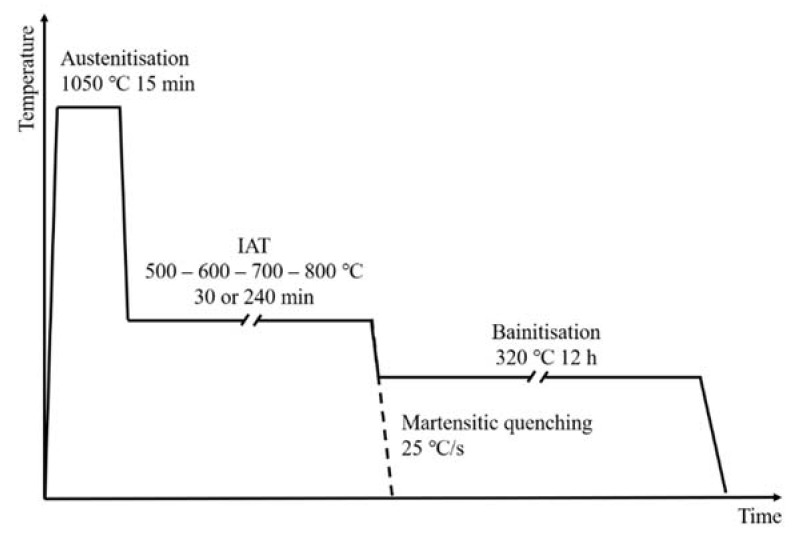
Scheme of conducted heat treatments with IAT. Variant with martensitic quenching was carried out to determine the M_s_ temperature changes after IAT (dashed line). A variant with isothermal bainitisation was made to discover the IAT impact on the bainitic transformation kinetics.

Two types of complex heat treatments were designed to achieve different phase transformations. They consisted of post-austenitising annealing segment—intermediate annealing treatment (hereinafter IAT)—followed by martensitic or isothermal bainitic quenching, as shown in Figure 1. The austenitising temperature and time for all variants were 1050 °C and 15 min, respectively. One of the higher austenitising temperatures was used for this steel. The parameters of this segment were selected to allow the entire ferrite volume to transform into austenite and to dissolve as many carbides as possible. After austenitising, cooling was applied to the IAT temperature at a rate of 25 °C/s. The temperature and time of IAT were 500, 600, 700, or 800 °C and 30 or 240 min, respectively, depending on the variant. The maximum duration of the IAT segment was chosen to avoid the pearlitic transformation occurring. After the stop, cooling was carried out at a rate of 25 °C/s to room temperature (martensitic quenching) or to a bainitising temperature of 320 °C (slightly above the M_s_ temperature). Bainitisation was led for 12 h. This relatively low temperature allowed the kinetics of bainitic transformation and the kinetics of carbide precipitation during bainitisation to be reduced. Because the IAT at 800 °C through 240 min increased the M_s_ temperature above 320 °C, and IAT at 700 °C for 30 min entered the pearlitic transformation area and likewise increased the M_s_ temperature, two additional heat treatments were performed. One consisted of IAT at 800 °C for 120 min, and the second consisted of IAT at 700 °C for 15 min 40 s (corresponding to a 45% advancement of pearlitic transformation incubation) to keep the M_s_ temperature low enough. The bainitising time used allowed a plateau for the bainitic transformation curve to be obtained. For IAT at 800 °C for 120 min the M_s_ temperature was about 320 °C and for other cases it was less than 320 °C. After soaking at the bainitising temperature, the samples were oven cooled. Moreover, the treatments without IAT were carried out as reference tests. The IAT parameters for all processes and their designations are included in Table 2.

All heat treatments were carried out using the DIL 805 L dilatometer (BÄHR Thermoanalyse GmbH, Hüllhorst, Germany). Cylindrical samples with a diameter of approx. 3 mm (4 mm for XRD analysis) and a length of approx. 10 mm were subjected to testing. An induction coil was used to heat the samples. Length changes were recorded using the LVDT (Linear Variable Differential Transformer) sensor. The tests were carried out under a vacuum. Inert gas (helium) was used as a cooling agent.

Light microscopy (LM), scanning electron microscopy (SEM), and transmission electron microscopy (TEM) observations were performed on specially prepared specimens. Observations were carried out on the cross-sectional surface of dilatometric samples. The LM samples were previously embedded in a conductive resin, sanded using 1200 grit abrasive paper, and then polished with a manual metallographic polishing machine (Norgpol Czerwiński S.J, Warsaw, Poland) using a diamond powder suspension with a particle size of 3 and 1 μm. To reveal the structure, Mi19Fe was used as the digestion agent. The LM observations were carried out with a Nikon ECLIPSE MA200 light microscope (Nikon, Tokyo, Japan). For SEM and TEM observations, samples were cut into thin slices, ground to a thickness of about 100 μm, and then electrolytically etched until their perforation. SEM observations were carried out with a Hitachi S-5500 scanning electron microscope (Hitachi High-Technologies Corporation, Tokyo, Japan). Additionally, the chemical composition for the chosen variants was investigated using an EDS detector on SEM. In this work, the average results that were determined based on tests in three places for each area are given. TEM observations were carried out with the transmission electron microscope TEM JEOL 1200 EX II (JEOL Ltd, Tokyo, Japan).

The phase composition of the steel samples that were subjected to different heat treatments was determined through X-ray diffraction (XRD). Measurements were carried out on a Siemens D500 diffractometer (Siemens, Karlsruhe, Germany) equipped with a high-resolution Si [Li] point detector that enabled the monochromisation of the Cu reflected beam, the maintenance of good measurement statistics, and a good signal/background ratio. For the apparatus, the radiation source was a Cu tube with operating parameters of U = 40 kV and I = 30 mA. The Bragg–Brentano θ/2θ measuring geometry was used with a measuring step of 0.02°. The measurements were performed on cross-sectional surfaces of dilatometric samples that were previously ground and polished, wherein the last polishing was carried out using a 100 nm Al_2_O_3_.

The kinetics of bainitic transformation for individual processes were described using Boltzmann sigmoid parameters fitted to the fragments of the dilatometric curves corresponding to the bainitising stage. The Boltzmann sigmoid equation in the adapted form is as follows:

(1) $$\Delta L=\frac{A_{1}-{\;A}_{2}}{{1+e}^{(t\;-{\;t}_{0.5})/\mathrm{dt}}}{+A}_{2}$$

where ΔL is the relative change in length, A_1_ is the minimal value on Boltzmann sigmoid, A_2_ is the maximal value on Boltzmann sigmoid, t is the time from isothermal hold start, t_0.5_ is the time in which half the sigmoid height is reached, and dt is the time constant.

**Figure 2:**
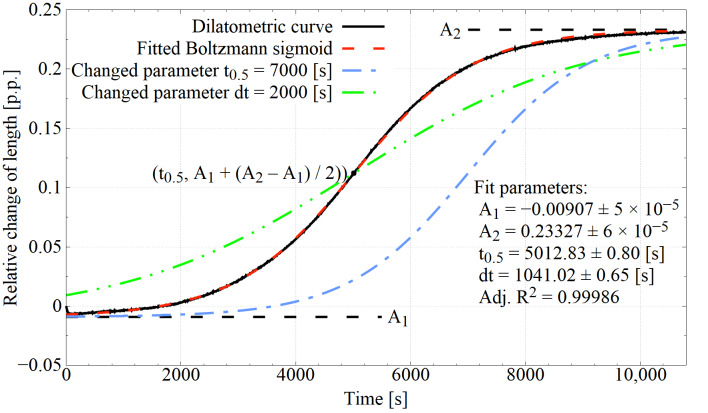
The use of Boltzmann sigmoids to represent the dilatometric bainitisation curve. Parameters of the sigmoids equation can be used to characterise the kinetics of bainitic transformation.

An example of a Boltzman sigmoid fitted to the experimental curve is shown in Figure 2. Parameters t_0.5_ and dt provide information on the kinetics of bainitic transformation. An increase in t_0.5_ causes the curve to shift towards higher t values on the abscissa relative to the original curve, which may be indirect information about the extension of the incubation time of the bainitic transformation and the initial substantial slowdown of transformation development. On the other hand, the increase in the value of dt causes the curve to be tilted towards the abscissa, with the rotation around the point with coordinates (t_0.5_, (A_1_ + (A_2_ − A_1_)/2)), which may provide information on the rate of bainitic ferrite autocatalytic growth.

## 3. Results

### 3.1. Dilatometric Study of IAT

**Figure 3:**
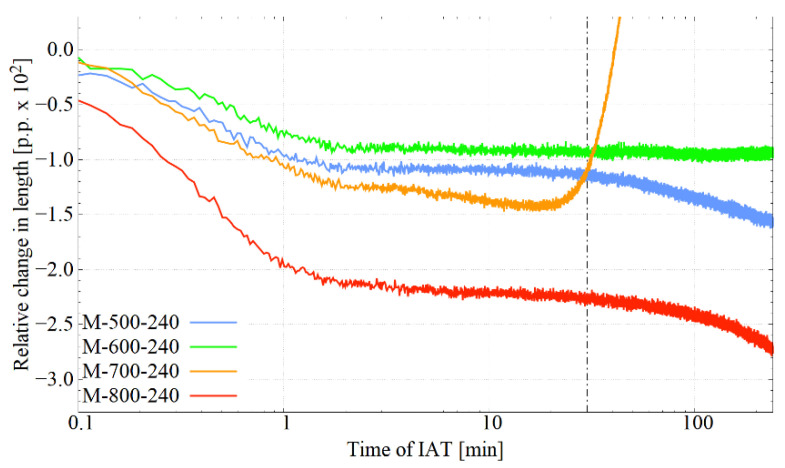
Dilatometric curves for the IAT segment. Visible decreases in length associated with the precipitation of carbides. During the IAT at 700 °C, there was entry into the range of pearlitic transformation.

Figure 3 presents the course of the dilatometric curves during IAT at the four considered temperatures. Their shape indicates the occurrence of phase transitions during the isothermal hold. The common feature for all curves is the initial section, where the decrease in sample length, lasting several minutes, can be observed. The most robust descent was observed for IAT at 800 °C, then at 700 °C, and for holds at 600 °C and 500 °C, which were similar. In the case of IAT at 800 °C, the decrease in length was not only the largest but also the tilt of the curve over the section in question was the highest, which indicates the intensity of transformation at this temperature. This behaviour of the dilatometric curve suggests the processes of carbide precipitation from austenite. The carbides released as phases with a lower thermal expansion coefficient than austenite caused a decrease in the sample length. Furthermore, depending on the IAT temperature used, one can notice a plateau (600 °C), a short plateau, followed by a further decrease in length (500 °C), a continuous mild decrease, intensifying with time (800 °C) and intensive growth (700 °C). The intensive growth at 700 °C indicates entry into the range of pearlitic transformation. At 700 °C, the formation of ferrite with a lower density than austenite caused an increase in the length of the sample. When comparing the curves presented, one should remember the component related to the difference in the heat expansion of the individual phases.

### 3.2. Bainitic Transformation Influenced by IAT

**Figure 4:**
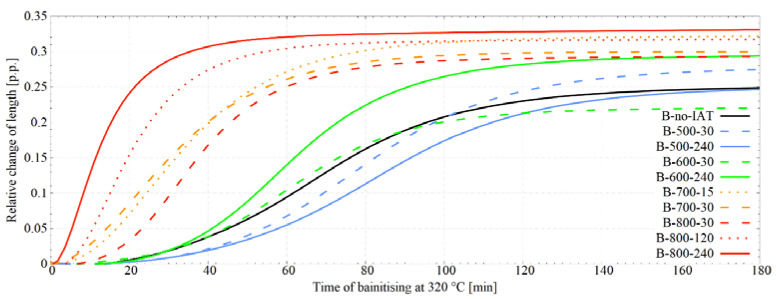
Dilatometric curves for the bainitising segment at 320 °C. Visible changes of bainitic transformation kinetics caused by the use of IAT—acceleration and deceleration of the transformation. Dilatometric curves were smoothed to minimise noise.

Figure 4 presents the dilatometric curves corresponding to the bainitisation stage at 320 °C that follows the IAT stage. Comparing the dilatometric curves representing the bainitic transformation after the IAT stage with the reference curve without this segment shows that the IAT appliance strongly influenced the kinetics of bainitic transformation. Furthermore, the way this impact manifested itself was dependent on the temperature and time of IAT. When the B-500-30 variant incubation stage was prolonged, the extinction stage started later, but after c.a. 100 min the initial transformation slowdown was compensated and eventually, a greater proportion of bainitic ferrite was obtained. The more prolonged IAT stage for the B-500-240 variant caused an even stronger weakening of the bainitic transformation kinetics, albeit after three hours the dilatometric curve was aligned with the reference curve without IAT. The B-600-30 variant entailed the slightest changes compared to the reference state, although the final bainitic ferrite content was lowered. Extending the hold time for the B-600-240 variant resulted in the acceleration of the transformation. For this variant, after c.a. 33 min, an even more intensive increase in bainitic ferrite share was observed, and ultimately more bainitic ferrite was obtained. It means that a longer duration of IAT brings an acceleration of the bainitic transformation rate. For the B-700-30 variant, a stronger acceleration effect was observed (even more robust than for B-800-30). However, in this case, an entry into the pearlitic transformation region occurred during the IAT. The dilatometric curve for this treatment was juxtaposed with the curve for the variant with 8 h IAT in the same temperature to determine the advancement of pearlitic transformation. Our analysis indicates that during the 30 min hold, the pearlitic transformation did not reach more than 0.8% advancement. However, to completely exclude pearlitic transformation for this temperature, the IAT time was shortened to 15 min 40 sec. For this variant (B-700-15), the bainitising curve was similar in shape to the B-700-30 variant initially, but after about 45 min, the curves diverged. For the B-700-15 variant, the bainitic transformation was still progressing, and ultimately more bainite was achieved than for the B-700-30 variant. An experiment with the 240 min IAT and bainitisation was not carried out due to the high amount of perlite formed during the IAT. For the B-800-30 and B-800-240 variants, the most substantial acceleration of bainitic transformation was obtained, wherein, as in the case of a hold at 600 °C, the fiercer effect occurred for a longer IAT time. Moreover, the B-800-30 variant allowed a share of bainitic ferrite similar to that obtained with the B-600-240 variant to be achieved. The strongest IAT effect is visible for the B-800-240 variant, where the largest volume fraction of bainitic ferrite was attained—much more than in the case of the reference treatment. Moreover, the bainitic transformation was significantly stimulated; the incubation stage was greatly reduced and the transformation developed very quickly and expired earlier than for other heat treatment variants. When comparing the dilatometric curve appropriate for the B-800-240 variant with the reference curve for the B-no-IAT variant, it is clear that by dint of IAT, the same bainitic ferrite content was obtained after 3 h without an IAT that was obtained after less than 20 min. It should be noticed that in the B-800-240 variant, bainitising was carried out slightly below the M_s_ temperature. The dilatometric curve for the B-800-240 variant was juxtaposed with the M-800-240 variant to determine the amount of martensite created. The contents of retained austenite and martensite were known for the quenched sample. The calculations showed that the amount of martensite formed prior to bainite was less than 0.1%. The bainitising curve after IAT at 800 °C but with a reduced time of 2 h (the B-800-120 variant) was similar in shape to the curve corresponding to IAT for 4 h hold but shifted towards longer times.

**Figure 5:**
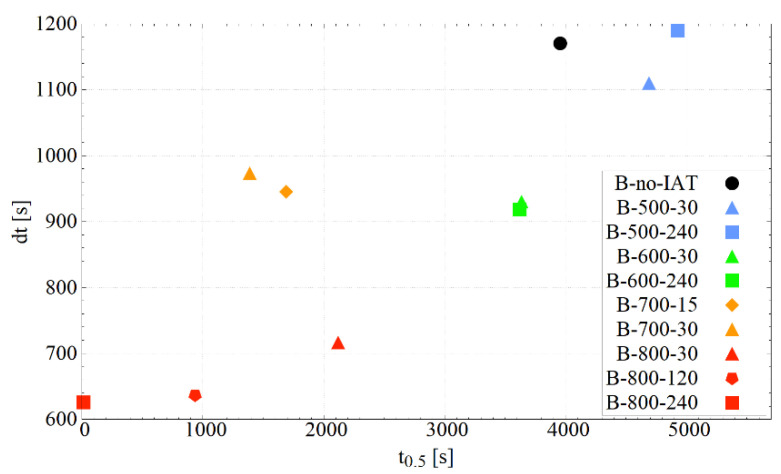
Representation of bainitisation dilatometric curves using Boltzmann sigmoid equation parameters. Parameter t_0.5_ corresponds to the time necessary to achieve 50% of the progress of transformation. Parameter dt describes the tilt of the curve—the higher it is, the more inclined the curve.

Boltzmann sigmoids were fitted to the obtained dilatometric curves. Table 3 lists the parameters of the matched sigmoids and the Adjusted R^2^. An excellent degree of fitting is visible. Figure 5 confirms that IAT use in all considered cases, except the B-500-240 variant, led to a decrease in the dt parameter, which indicates the acceleration of the bainitic transformation due to IAT. In addition, there was a tendency to reduce dt as the IAT temperature increases. Moreover, a decrease in parameter t_0.5_ was observed in all cases (except the B-500-30 and B-500-240 variants), which indicates that the application of IAT allows 50% of the bainitic transformation (specific value for a given temperature) to be achieved earlier. It can be seen that the increase in the IAT temperature correlates with the decrease in the value of the t_0.5_ parameter. The results for the B-700-30 variant clearly deviate from the trends presented. This is not a consequence of a small entry during the isothermal hold into the range of pearlitic transformation. For the B-700-15 variant (with no pearlitic transformation), a similar result was obtained. The results suggest that the time of IAT duration has a considerable effect on both the dt and t_0.5_ parameters. However, more measuring points would be needed to confirm this relationship.

### 3.3. Microstructural Changes

#### 3.3.1. X-ray Diffraction

**Figure 6:**
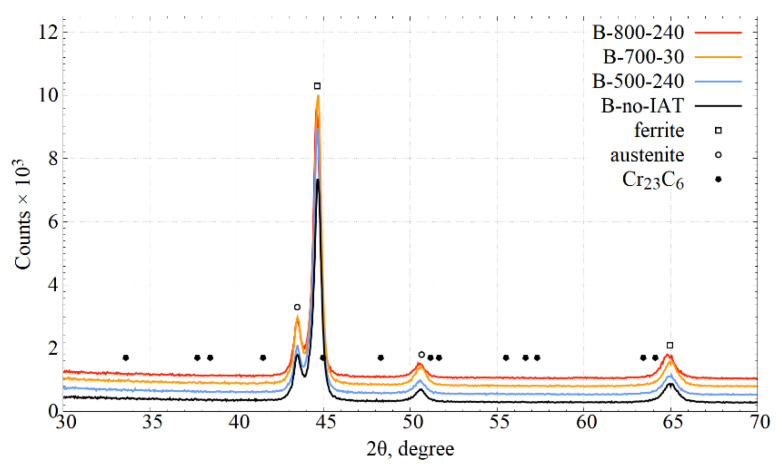
XRD patterns of the X37CrMoV5-1 after different heat treatments. The experimental results suggest a two-phase composition: ferritic–austenitic.

Figure 6 presents the XRD patterns obtained for selected variants of bainitising treatments. Markings of ferrite and austenite peaks and pointing potential signal locations for Cr_23_C_6_ carbides were applied. Individual XRD spectra were shifted vertically to increase readability.

Peaks from the austenite (retained) and ferrite (bainitic and martensitic) crystals are clearly visible. Comparing their height relative to individual treatment variants is burdened with a significant error resulting from the martensitic transformation during cooling after bainitising. Because of the changes in the kinetics of the bainitic transformation, the kinetics of martensitic transformation also changed. Because martensitic transformation ultimately determined the phase composition of the steel system, the peak heights do not follow the phase composition immediately after bainitising. Signals from carbides were not noticed for any of the treatments carried out. The absence of reflections on experimental diffraction patterns from the carbide phases was probably due to the small size of the crystallites of these phases and/or the high density of defects.

The measured diffractograms do not show the effect of ferrite tetragonalisation as a result of martensitic transformation. This is probably because the magnitude of this deformation was below the resolution of the diffraction method.

#### 3.3.2. Light Microscopy

**Figure 7:**
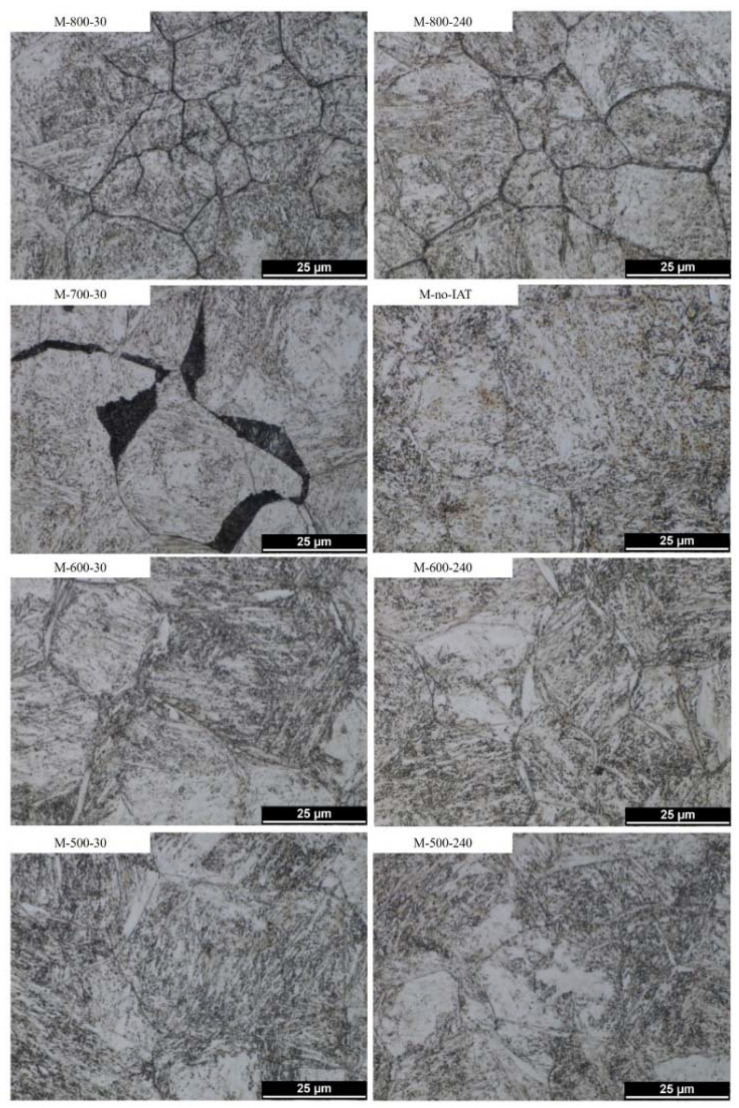
LM microstructures of martensitic quenched steel with a different variant of IAT or without it. More intensive PAGBs etching is visible for IAT variants.

**Figure 8:**
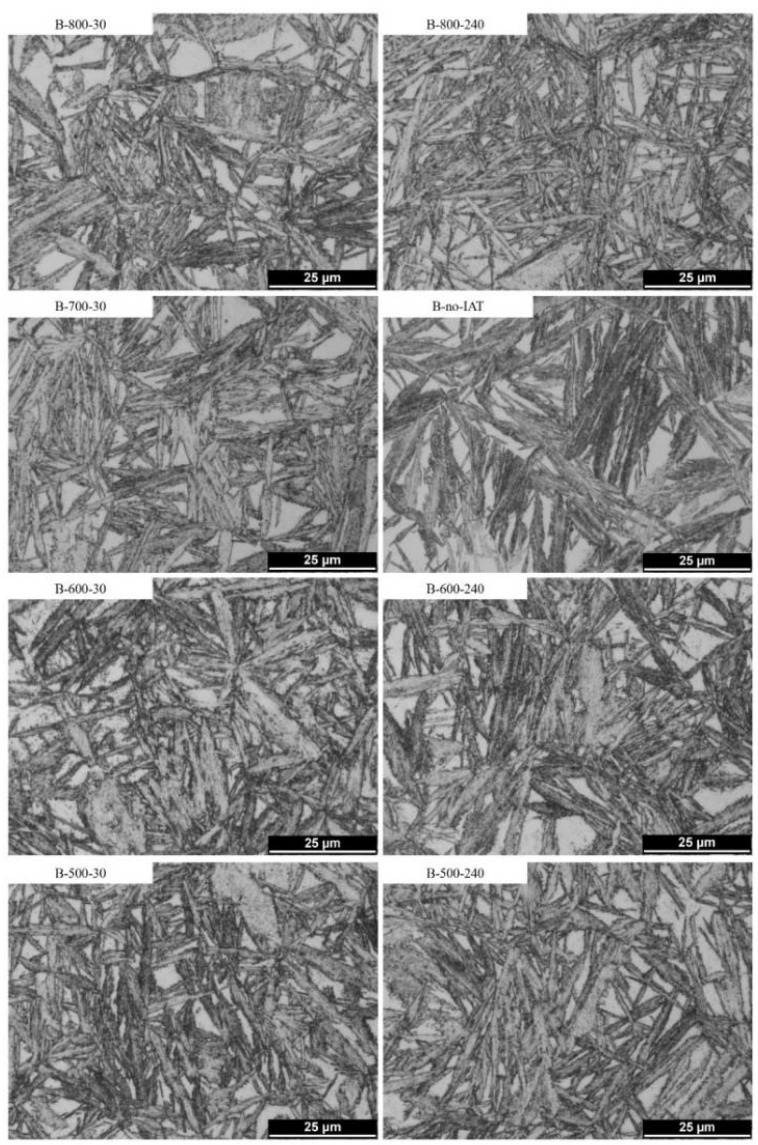
LM microstructures of bainitised steel with a different variant of IAT or without it. A stronger microstructure fragmentation is visible for the IAT variants.

The microstructures obtained for individual variants of martensitic quenching and bainitisation are shown in Figure 7 and Figure 8, respectively. As can be seen, in the case of martensitic quenching—Figure 7—a stronger etching of the prior austenite grain boundaries (PAGBs) was observed for variants containing IAT than for the variant without IAT. This indicates a change in the chemical or phase composition at PAGBs. The strongest PAGBs etching occurred with IAT variants at 800 °C; they were also noticeable at lower temperatures, although not quite as pronounced. For the M-700-30 variant, extensive dark-etching areas with a flat interface relative to one PAG and irregular to the other grain were visible. PAGBs decorations were no longer visible in steel microstructures after bainitising treatment.

Figure 8 depicts the microstructures obtained through bainitising treatments that were or were not preceded by the IAT segment. The microstructures contained different fractions of the bainitic ferrite (dominant), martensite (formed during cooling after bainitising), retained austenite (bright areas), and fine carbides. It can be seen that the use of IAT promotes the refinement of the microstructure. The refinement results from the formation of thinner and shorter bainite sheaves, which are differently oriented towards each other and are often interlocking, separate areas of austenite. The strongest refinement was visible when using IAT at 800 °C, particularly for the B-800-240 variant.

Moreover, areas suggesting the presence of acicular ferrite are visible for microstructures obtained through treatments using IAT—Figure 8 (especially B-800-240).

#### 3.3.3. Transmission Electron Microscopy

**Figure 9:**
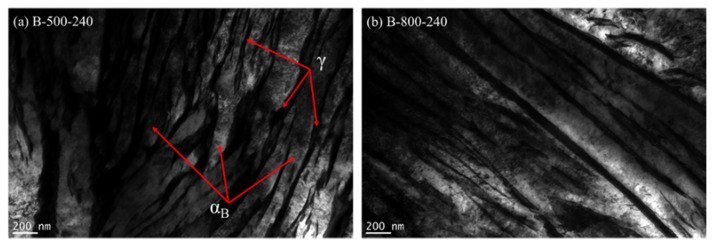
TEM microstructures of bainitised steel using extreme variants of the IAT: (**a**) at 500 °C by 240 min, (**b**) at 800 °C by 240 min. Nano-areas formed by bainitic ferrite (light, α_B_) and thin layers of residual autenite (dark, γ) are visible.

TEM observations were carried out for variants with bainitisation using extreme conditions of IAT—B-500-240 and B-800-240. The conducted investigation showed the presence of nanobainite areas obtained by performed treatments. Figure 9 shows nanostructures consisting of bainitic ferrite plates (light areas) separated by thin layers of residual austenite (dark areas). The thicknesses of bainitic ferrite plates and austenite layers determined based on measurements from 50 randomly selected places were, respectively: 63.5 ± 42.5 and 53.4 ± 32.5 nm for the B-500-240 variant and 76.3 ± 36.5 and 40.23 ± 11.7 nm for the B-800-240 variant.

#### 3.3.4. Scanning Electron Microscopy and Energy Dispersive X-ray Spectroscopy

**Figure 10:**
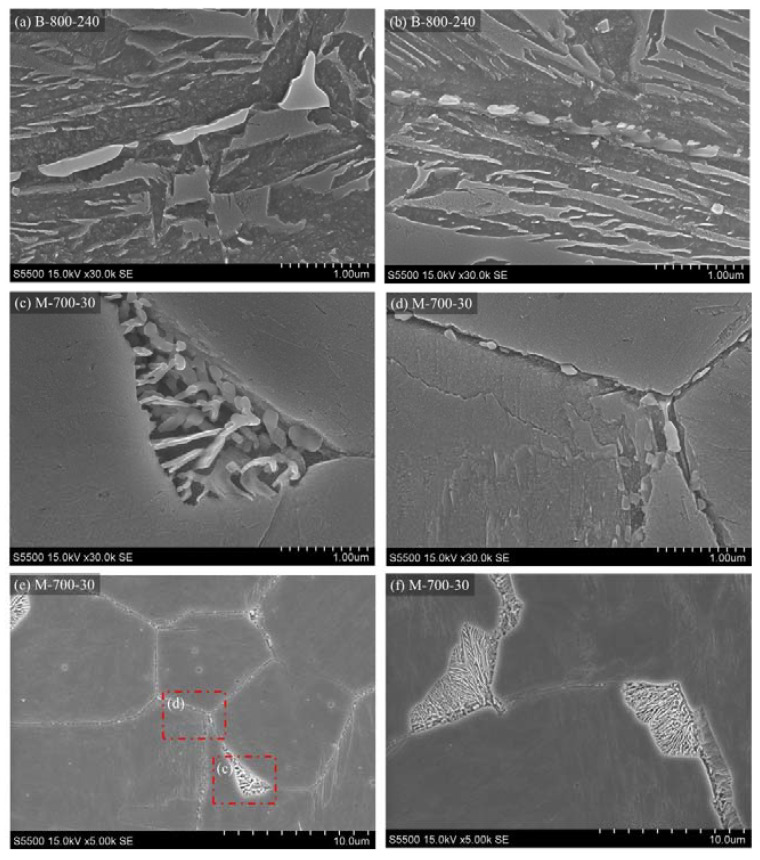
SEM microstructures of steel samples after selected treatments: (**a**,**b**) bainitisation preceded by IAT at 800 °C by 240 min, (**c**–**f**) martensitic quenching preceded by IAT at 700 °C by 30 min.

SEM observations and EDS analysis allowed changes in the chemical composition in steel caused by phase changes occurring during IAT to be determined. The obtained images for the chosen variants are shown in Figure 10. The use of an electron microscope made it possible to notice the previously invisible precipitations. They are located on the PAGBs in regions identified by the LM as dark-etching areas. The morphology of the precipitations depends on the IAT conditions. In the B-800-240 variant, the PAGBs are decorated discontinuously, and the precipitations have irregular shapes. For the M-700-30 variant, precipitations similar to those obtained for B-800-240 can be seen, as well as extensive areas with lamellar structures.

It can be seen that the flat interface is maintained by precipitations relative to one of the prior austenite grains (PAGs) and irregular growth into the other austenite grains. For the variant with IAT at 500 °C, no similar precipitations were observed.

**Figure 11:**
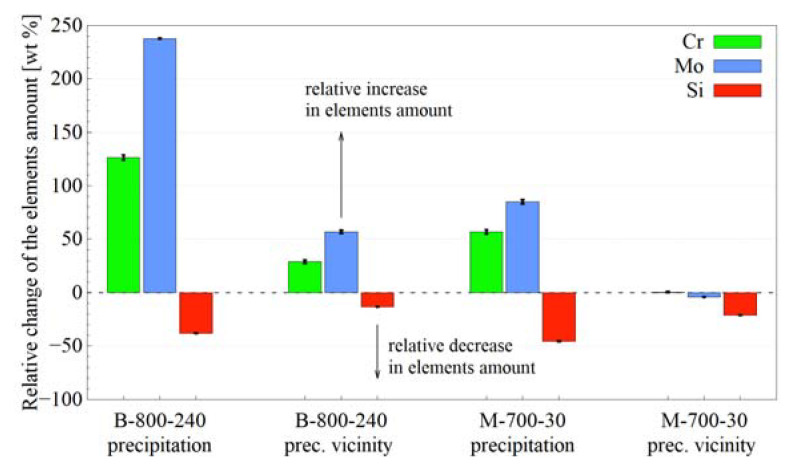
Relative change in the amount of the element according to the EDS analysis for precipitations and the vicinity of the precipitations in relation to the PAGs interior.

Individual areas of microstructures—precipitations at PAGBs, vicinity of precipitations and PAGs interiors—were subjected to EDS analysis. Figure 11 presents a change in the amount of the elements in selected areas related to the PAGs interior. It can be seen that the precipitations show a higher amount of chromium and molybdenum and a lower amount of silicon when compared to the interior of PAGs. The most substantial effect was seen for B-800-240. The differences in the amount of elements in the vicinity of the precipitations in relation to the PAGs interior were also visible; however, they were weaker. For the B-800-240 variant, the effect is similar to that for the precipitations, i.e., chromium and molybdenum enrichment and silicon depletion. For the M-700-30 variant, the changes in the amount of chromium and molybdenum were slight; however, they were significant for silicon.

## 4. Discussion

This study revealed that IAT affects the subsequent bainitic transformation in the X37CrMoV5-1 steel. Three types of changes made by IAT on the bainitic transformation kinetics can be distinguished. The first type is related to the incubation time, the second to the autocatalytic transformation stage, and the third to the final bainitic ferrite share. Understandably, the direction and intensity of these changes depends on the temperature and the time of IAT, which determine the phenomena occurring during this stage.

### 4.1. Phase Transformations during IAT

It was confirmed during the IAT process that various phase transformations had occurred. The course of the dilatometric curves clearly demonstrates this. The decrease in the sample length indicated the precipitation of a phase or phases for which the crystal lattice, at the given temperature (effect of thermal expansion), was denser than the austenite lattice. In the present case, these were the carbide phases.

The most intense precipitation took place at the beginning of the IAT during the first few minutes, regardless of the temperature variant—Figure 3. The further decrease in length can be interpreted as the start of carbide precipitation at other locations or the precipitation of different carbide types, or the growth of existing ones. This effect was also confirmed by the increase in the M_s_ temperature observed during cooling after the individual IAT variant. The rise of the M_s_ temperature indicated a depletion of austenite in carbide forming elements such as carbon or chromium.

A stronger etching of the PAGBs after martensitic quenching preceded by IAT indicated their decoration with precipitations—Figure 7. LM observations also indicated their morphological diversity. In the case of IAT at 800 °C, the presence of precipitations caused a strong etching of the PAGB. For IAT at 700 °C, the effect of etching was weaker. There were extensive darkly etched areas that adhered smoothly to one PAG and often had an irregular interface relative to the other. For IAT at 600 °C and 500 °C, PAGBs etching was the weakest but was still more robust than for the variant without IAT. It seems that the described differences depend on the temperature and time of IAT, which control the kinetics of carbide precipitation and growth.

The XRD analysis showed only ferrite and austenite: no other phases were detected—Figure 6. The imperceptibility of carbides during the XRD analysis does not contradict their occurrence. It may indicate a very small crystallite size below the detection capacity of the method or their insufficient volume fraction. However, SEM observations showed the presence of precipitations on PAGBs—Figure 10. The EDS analysis indicated that they were rich in chromium and molybdenum when deficient in silicon—Figure 11. This suggests that the precipitations may be carbides made of these metallic elements.

**Figure 12:**
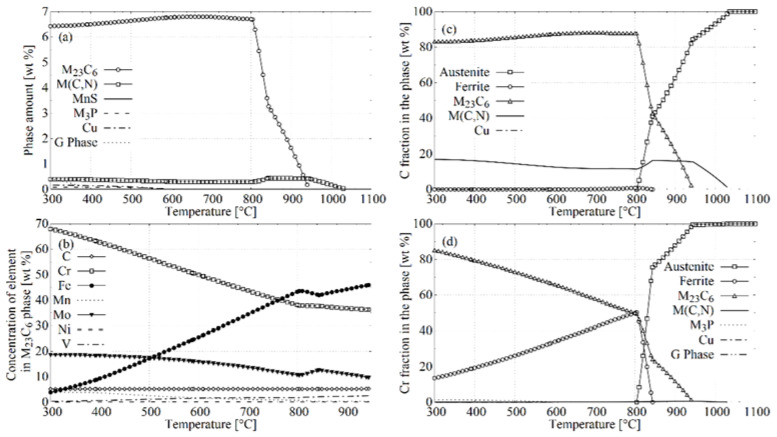
Equilibrium (**a**) phase composition; (**b**) chemical composition of the M_23_C_6_ phase; (**c**) phase distribution of C atoms; (**d**) distribution of Cr atoms in phases for the X37CrMoV5-1 steel according to the JMatPro simulation.

Figure 12 summarises the results of the simulations carried out using the JMatPro software (Sente Software Ltd., Guildford, UK, version 9.1). Figure 12a shows the equilibrium content of the precipitations in the X37CrMoV5-1 steel. As can be seen, M_23_C_6_ carbides, which occurred in the system below 930 °C, constitute the dominant equilibrium type of precipitation. Their maximum equilibrium weight fraction fell at a temperature of 670 °C and was less than 6.8 wt %. Below this temperature, it decreased to a value of just over 6.4 wt % at 300 °C. The second type of precipitations—M(C, N)—occurred in the temperature range of 1030–300 °C. Their content was in the range of 0.03–0.41 wt %, which as significantly lower than for the M_23_C_6_ carbides. The content of other equilibrium precipitations simulated with JMatPro software was even lower. Thus, computer simulations suggested that the M_23_C_6_ type carbides were the dominant type of precipitation in the equilibrium. Although the treatments carried out using IAT did not allow equilibrium to be obtained, it is obvious that the system strove to achieve this state.

The M_23_C_6_ carbides (M = Fe, Cr, Mn, Mo, Ni and V) are the secondary carbides present in many steel grades if the chemical composition and heat treatment conditions allow their occurrence. Much research has been carried out on this kind of carbide in austenitic stainless steels where they can cause intergranular corrosion [26]. Although the X37CrMoV5-1 steel is not austenitic steel, the conclusions drawn from the studies mentioned are very valuable for the current research. They can help the observed phenomena to be understood. During IAT, the carbides nucleate in the austenitic matrix.

M_23_C_6_ carbides crystallise in the FCC structure. The ratio of their parameters and the austenite lattice parameters is 3:1 [27]. In austenitic stainless steels they nucleate predominantly at general austenite grain boundaries (AGBs) [28,29,30], at incoherent twin boundaries [29,31,32], but also intra-granularly on dislocations [28,33,34] or on the MX (M = Ti, V, Nb; X = C, N) precipitations [29,35,36]. They can also nucleate homogeneously at high temperatures [37]. In addition, they can be formed by rebuilding other types of carbides (e.g., M_3_C) [38] and can also be a product of the pearlitic transformation of austenite [39]. At the general AGBs, the M_23_C_6_ carbides nucleate in a cube–cube orientation relationship forming a coherent interface with one of the austenite grains which connect at the boundary [27,34]. In the second neighbouring grain, the carbide growth occurs incoherently deep into this grain [27,30,40]. Thus, precipitation at AGBs forms a smooth, flat, and coherent interface with one austenite grain and an irregular, wavy, and incoherent interface with the second grain [27,41]. It should be noticed that carbides do not have to nucleate evenly on all available grain boundaries, but their nucleation depends on the structure of individual boundaries and the misorientation between grains [32,37]. This particular morphology of the precipitations was revealed during the LM and SEM observations. Under appropriate conditions, due to the precipitation and growth of carbides at the AGBs, lamellar carbides may form [34] consisting of many connected carbide precipitations [42]. As reported by Wang et al., the addition of silicon to steel can affect the mutual orientation of M_23_C_6_ carbides [43]. Moreover, M_23_C_6_ carbides formed inside the austenite grain lose coherence with the austenitic matrix during their growth [44].

The simulations of changes in the chemical composition of M_23_C_6_ carbides are presented in Figure 12b. In addition to carbon, which is their obvious component, they are also made of metallic elements such as Fe, Cr, Mn, Mo, Ni, and V, with the first three being the main components of carbide. The equilibrium chemical weight composition of M_23_C_6_ carbide at 940 °C was as follows: 45.76% Fe, 36.28% Cr, 9.68% Mo, 5.25% C, the rest is Mn, Ni, and V. With a decrease in temperature, the Fe content in this type of carbide decreased, while the Cr and Mo content increased. The carbon concentration remained practically unchanged. At 800 °C the composition was as follows: 43.23% Fe, 38.13% Cr, 10.66% Mo, 5.24% C; at 700 °C it was already: 34.41% Fe, 43.70% Cr, 13.62% Mo, 5.19% C; at 600 °C: 25.60% Fe, 49.90% Cr, 15.88% Mo, 5.17% C; and at 500 °C: 17.19% Fe, 56.40% Cr, 17.39% Mo, 5.16% C. Thus, as the temperature decreased, the Cr and Mo concentrations increased, while the concentration of Fe decreased.

Changes in the amount of M_23_C_6_ carbide in the system and changes in its chemical composition entailed changes in the distribution of elements inside the system—Figure 12c,d. As the temperature decreased, the concentration of carbon atoms in M_23_C_6_ carbide increased, while its concentration in austenite decreased. Below 800 °C, more and more chromium atoms accumulated in carbides at the expense of the depletion of ferrite. The experiment confirmed the enrichment of carbide areas in chromium. The growth of M_23_C_6_ carbides was accompanied by a drainage of chromium atoms from the carbides’ vicinity [30,45,46,47,48]. Due to the much lower diffusivity of Cr atoms compared to the diffusivity of carbon atoms, the diffusion of the former element controls the growth of M_23_C_6_ [49]. At higher temperatures, where bulk diffusion is possible, drainage can significantly affect the interior of the grain into which the carbide grows. The mentioned drainage can also occur along the APGs, which are fast diffusion pathways [27] that act as collector plates to supply the growing M_23_C_6_ [48].

As noted by Hall and Briant, the depletion at the AGBs in chromium is more substantial at lower temperatures and at higher temperatures (or longer soaking times). The depletion profile observed across the grain boundary is shallower but broader [50]. They also noticed the asymmetry of the depletion profiles associated with the discontinuous release of M_23_C_6_ carbides on one side of the grain boundary and the depletion in chromium along the AGBs. The depletion they observed along the grain boundaries was larger by one order of magnitude than the depletion into the grain; it was observed even about 3 microns from the separated carbide. Kaneko et al. also pointed out the depletion in chromium along the grain boundaries due to M_23_C_6_ precipitation [30]. Along with the lowering of the temperature, the diffusion across grain boundaries becomes more important. As the results of the research showed, it was not only chromium atoms that were drained, but molybdenum atoms as well [50,51]. If the temperature allows for a diffusion of substitutional atoms, iron atoms are replaced by chromium [52] or molybdenum [51] in carbides. The mentioned exchange of atoms leads to the expansion of carbides lattice parameter and the coarsening of these carbides [53]. Consequently, this may lead to the corrosion of the areas close to PAGBs, which is manifested during chemical etching while revealing the microstructure. This explains the stronger etching of PAGBs when using IAT, especially for the M-800-30 and M-800-240 variants—Figure 7.

### 4.2. Impact of IAT on Incubation of Bainitic Transformation

Our research showed that the application of IAT at 800 and 700 °C allowed a significant reduction in the incubation time of the bainitic transformation in the X37CrMoV5-1 steel.

The incubation time of bainitic transformation is necessary for the formation of stable nuclei of bainitic ferrite. According to the displacive phase transformation concept, the bainitic transformation in steel occurs due to coordinated movements of iron and substitutional atoms as a result of deformation with an invariant plane and a large shear component [54,55,56]. Thermal vibrations of the crystal lattice may lead to slight shifts of the lattice planes, in which a limited number of iron and substitutional atoms is involved. It happens without diffusion and without changing the chemical composition relative to the parent austenite phase. It leads to a change in the iron structure from FCC to BCC in micro-areas. Spontaneous dissociations of dislocations already present in the parent austenite may lead to their development in the nuclei of austenite transformation into ferrite. The places of the first nucleation can be AGBs, inclusion surfaces, or dislocations [57]. In this way, the first subunits of bainitic ferrite are created.

The precipitation of carbides during IAT leads to an impoverishment of the areas at the AGBs in alloying elements such as chromium, molybdenum, and carbon, which are needed for carbide growth. These alloying elements cause the expansion of the austenite lattice. Drainage of these elements to carbides leads to the austenite lattice contraction, which would promote the transformation of the undercooled austenite into bainitic ferrite similar to the effect of vacancies suggested by Liu et al. [58]. It means that the depletion of the austenite lattice in atoms of these elements would increase the likelihood of Bain transformation in small volumes. In other words, it would facilitate the formation of bainitic ferrite nuclei.

The influence of many alloying elements on the kinetics of bainitic transformation is well known. Work by Catteau et al. showed that the increase in the concentration of carbon atoms leads to an extension of the incubation time of bainitic transformation and a deceleration of the transformation kinetics and a reduction in the volume of the produced bainitic ferrite [59]. The results of the research by Deschère and Quidort showed that a decrease in the amount of chromium promotes a more significant share of bainitic ferrite at the end of the transformation, as well as an acceleration of its kinetics [60]. A similar effect was observed for molybdenum [61]. Furthermore, Quidort and Brechet showed that steels containing a carbide-forming element but with reduced silicon are characterised by faster kinetics of bainitic transformation at a given temperature and a higher final amount of bainitic ferrite [62].

The results of our research suggest that the effectiveness of using IAT to reduce the incubation time and accelerate the kinetics of bainitic transformation is determined by the temperature and time of IAT. These parameters affect the kinetics of carbide precipitation and growth as well as the chemical gradient of alloying elements in the parent austenite. This is due to the diffusive nature of these processes. This would explain the strong reduction in incubation time due to IAT at 800 and 700 °C, as well as the slight decrease due to IAT at 600 °C. However, this does not explain the incubation time extension of the bainitic transformation observed for the IAT variant at 500 °C compared to the heat treatment without IAT. The performed experiments and computer simulations indicate that during IAT, the precipitation of M_23_C_6_ carbides occurred. Thus, after IAT at 500 °C, one should expect a reduction in the incubation time, which would be a similar albeit weaker than after IAT at 600 °C. The diffusion processes that occur during IAT can lead to the formation of a chemical gradient associated with the growth of precipitated carbides and the homogeneity of the chemical composition inside the parent austenite grains. Local heterogeneity in the chemical composition resulting from the dissolution of carbides during austenitisation, which has not been previously eliminated, may be compensated during IAT at 500 °C. Homogenisation of the chemical composition, mainly the carbon and chromium concentration, leads to the elimination of small depletion areas, which could provide favourable nucleation sites of ferritic bainite in the early stages of transformation. Thus, a relatively low-temperature IAT would lead to the homogeneity of the chemical composition.

An additional aspect to mention is that the greater the undercooling, the more nuclei of the precipitations and the smaller their critical radius. IAT dilatometric analysis clearly showed that at 500 °C, precipitation processes occurred, although LM and SEM observations did not allow such evident effects of carbides to be seen as for IAT at 800 or 700 °C. It is likely that many fine carbides formed along PAGBs. M_23_C_6_ carbides can nucleate alternately on both sides of the PAGB. While from the grain side on which the carbide nucleates, towards which it maintains coherence, there should be no impediment to the nucleation of bainitic ferrite (due to the high crystallinity similarity of M_23_C_6_ to austenite [63]), the nucleation of the bainitic ferrite from the grain side in which the carbide incoherently increases can be hindered. Nucleating bainite must maintain a K–S orientation with the austenite grains on both sides of the grain boundary [64]. This can be difficult with the dense distribution of fine carbides and the small free spaces between them. In addition, the presence of M_23_C_6_ precipitations leads to mismatched dislocations on the M_23_C_6_–austenite interface, which are caused by a mismatch of the lattices [30], which may also affect the nucleation of the bainitic ferrite. As noted by Guo et al., in the case of allotriomorphic ferrite at PAGBs, the maintenance of a K–S orientation with the matrix led to the promotion of bainitic ferrite nucleation because the nuclei were able to create a low-energy interface with austenite [65]. In the absence of proper orientation between allotriomorphic ferrite and austenite, the bainitic ferrite nucleation was not observed. A similar situation may occur with the application of the IAT stage. Moreover, the research and considerations of Gomez et al. should be referenced here. As indicated by their work, the greater the curvature of the surface that is the site of heterogeneous nucleation, the greater the radius of the critical nucleus of the emerging phase [66]. In the case of PAGs covered with fine precipitations (large surface curvature), the size of the critical nuclei of the bainite transformation would be significantly increased. These factors can reduce the density of the bainitic ferrite nuclei, as well as reduce the likelihood of their appearance. Hence, on the map representing the sigmoidal fit of the dilatometric curves—Figure 5—there was a strong tendency to decrease the value of the t_0.5_ parameter with the increase in time and temperature of the IAT for variants at 600, 700, and 800 °C, as well as the opposite effect in the case of IAT at 500 °C. The location of points representing variants with IAT at 700 °C outside the line demarcated by the remaining points corresponding to IAT is very intriguing. This indicates the occurrence of additional effects that should be examined in further research.

We conclude that from a thermodynamic point of view, the effect of IAT on the incubation of bainitic transformation in the X37CrMoV5-1 steel is twofold and consists of changing the density of potential grain boundary nucleation sites and the activation energy for grain boundary nucleation. The total nucleation rate during bainite formation from a fully austenitic phase, $\frac{\mathrm{dN}}{\mathrm{dt}}$, was given by Ravi, Sietsma and Santofimia [57]:

(2) $$\frac{\mathrm{dN}}{\mathrm{dt}}={\left(\frac{\mathrm{dN}}{\mathrm{dt}}\right)}_{G}+{\left(\frac{\mathrm{dN}}{\mathrm{dt}}\right)}_{A}$$

where ${\left(\frac{\mathrm{dN}}{\mathrm{dt}}\right)}_{G}$ is the nucleation rate per unit volume due to nucleation at austenite grain boundaries and ${\left(\frac{\mathrm{dN}}{\mathrm{dt}}\right)}_{A}$ is the nucleation rate per unit volume due to autocatalytic nucleation. In their work, they propose the expression ${\left(\frac{\mathrm{dN}}{\mathrm{dt}}\right)}_{G}$ using the following formula:

(3) $${\left(\frac{\mathrm{dN}}{\mathrm{dt}}\right)}_{G}=\frac{\mathrm{kT}}{h}N_{\mathrm{tG}}\exp\left(-\frac{Q_{G}^{*}}{\mathrm{kT}}\right)$$

where k is the Boltzmann’s constant, h is the Planck’s constant, $N_{\mathrm{tG}}$ is the number density of potential grain boundary nucleations sites at given time t, $Q_{G}^{*}$ is the activation energy for grain boundary nucleation, and T is the isothermal transformation temperature. The precipitation of M_23_C_6_ carbides during IAT would therefore lead to a decrease in $N_{\mathrm{tG}}$, which would reduce the degree of bainite nucleation, and at the same time, could cause a reduction in $Q_{G}^{*}$ resulting in the opposite effect. The predominance of one of the competing factors over the other would be determined by the morphology and distribution of carbides, as well as the kinetics of the diffusion processes leading to the formation of a chemical composition gradient or its homogeneity. These factors, in turn, depend on the IAT parameters.

In their work, Ravi et al. [23] studied the effect of IAT on bainitic transformation in steel containing 0.2 wt % C. They suggested that during IAT, the nuclei of ferrite may be formed, which during bainitising at lower temperatures may develop into bainitic ferrite. This would be facilitated by a change in the chemical composition occurring during the IAT, resulting from the segregation of carbon atoms into PAGBs. Thanks to this, there would be an increase in the density of bainite nucleation sites and an acceleration of transformation. Their research indicates that the lower temperature and longer time of IAT intensifies this acceleration. Another effect was revealed in the present study: with the lowering of the IAT temperature, the acceleration of bainitic transformation weakened. Moreover, in samples of the B-500-240 variant, the bainitic transformation rate was slower than in the samples without IAT (B-no-IAT). The differences between both research works can be explained by the differences in the processes occurring during the IAT. In the aforementioned work, the authors observed during the IAT the segregation of carbon atoms into PAGBs and the formation of ferrite nuclei. In the present study, the precipitation of carbides took place during IAT. The morphology and distribution of precipitations were more favourable for IAT at higher temperatures, as well as the diffusion processes, the impact of which was also more favourable at higher IAT temperatures.

In another work, Ravi et al. [22] concluded that in the case of steel containing 1.05 wt % C due to the formation of cementite during IAT, the bainitic transformation is accelerated through the faster onset of bainite formation. The formation of small, isolated regions of GB cementite, as well as an increase in these precipitates, and an increase in these precipitations would lead to the decrease in activation energy for the bainite nucleation process. This happens through the drainage of carbon atoms near the cementite–austenite interfaces, creating favourable thermodynamic conditions for heterogeneous nucleation. They also suggested that cementite–austenite interfaces could provide additional sites for heterogeneous nucleation, increasing $N_{\mathrm{tG}}$. Our current knowledge does not allow us to agree with their last postulate: the work to date devoted to ferrite nucleation on various secretions and inclusions has not shown that cementite is an effective nucleation site for ferrite, including bainitic [67]. As noted by Ravi et al. [22], the decrease in the IAT temperature was accompanied by an increase in the amount of cementite in the microstructure as well as a change in morphology; for IAT at 800 °C, they observed a continuous cementite layer.

### 4.3. Impact of IAT on the Autocatalytic Phase of Bainitic Transformation

The experiments have shown that IAT use affects the autocatalytic bainitic transformation period, which is manifested by a change in the tilt of the dilatometric curve. This research indicates that as the temperature and time of IAT increased, the intensity of autocatalytic growth of bainitic ferrite sheaves increased as well. However, for the B-500-240 variant, the opposite effects were observed.

**Figure 13:**
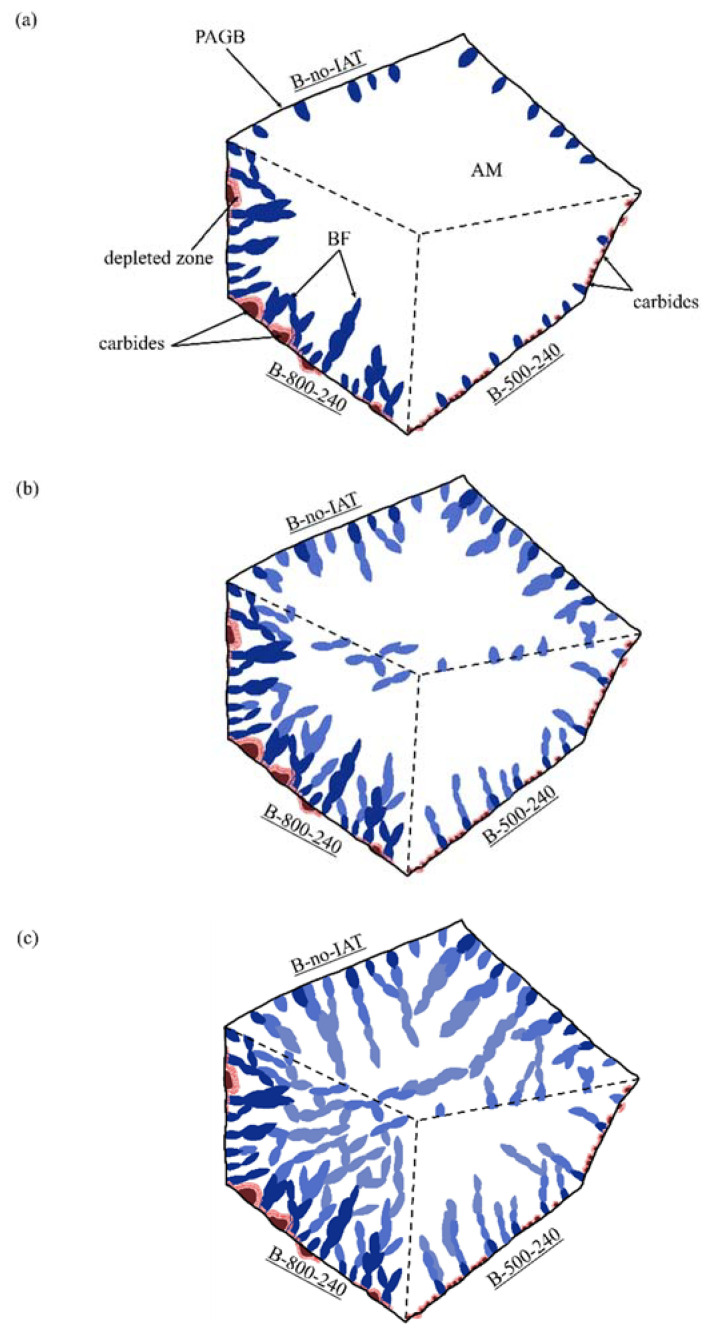
Schematic representation of the impact of IAT on bainitic transformation. (**a**) the presence of carbides on PAGBs may promote the nucleation of bainitic ferrite (BF) or impede it; it depends on their concentration, morphology, and distribution; (**b**) the amount of bainite subunits formed in the first stage directly affects the autocatalytic growth of the sheaves; (**c**) by adequately selecting the IAT parameters it is possible to shape the final bainite microstructure.

The growth of bainitic ferrite subunits is completely diffusionless and occurs rapidly due to the displacive mechanism [54]. The deformation of the crystal lattice caused by the formation of subunits prevents their further growth, which is blocked by the stress associated with the deformation of the area undergoing transformation. Blocking the growth of subunits causes new nucleations, which allow the transformation to continue [68]. Immediately after the formation of subunits, due to the lower solubility of carbon in ferrite than austenite, excess carbon is ejected from the subunits [69]. Bainite formation is continued by the autocatalytic nucleation of bainitic ferrite on the newly formed subunits. New subunits usually nucleate more frequently near the sharp edges of other bainitic ferrite plates than on their flat surfaces [68,69]. The blocking of the growth of subsequent subunits is due to the stress field around the plate, a collision with other plates, or with PAGBs [70]. The size of the subunits depends on the available austenite, in which nucleation occurs; it is therefore limited and is still significantly smaller than the size of the austenite grain. The observed differences in the course of dilatometric curves for bainitisation were caused by changes in bainitic ferrite nuclei density at the initial stage of transformation. The explanation of the differences in nuclei density has already been proposed in the previous section. The more nuclei that are present, the more subunits are formed, providing space for subsequent heterogeneous nucleation and faster sheave formation, as shown schematically in Figure 13. The development of bainitic transformation from numerous places gives, in turn, a more fragmented austenite grain—Figure 9—and a larger final share of bainitic ferrite. Because of the accelerated kinetics of bainitic transformation, large volumes (blocks) of austenite are not excluded from the transformation. Thus, the incomplete transformation phenomena related to carbon enrichment is weakened. We observed such behaviour for treatments with IAT at 800, 700, and 600 °C. The more sheaves that were present, the closer to one another they could form, despite the progressive enrichment of the remaining austenite in carbon.

Changes in the kinetics of bainitic transformation induced by IAT and associated with the stage of autocatalytic growth are shown in Figure 5. A strong tendency to decrease the value of the sigmoid dt parameter with the increase in time and temperature of the IAT for variants at 600, 700, and 800 °C can be observed. Moreover, the opposite effect in the case of IAT at 500 °C is visible. Moreover, in this case, the points corresponding to IAT at 700 °C deviated from the expected position.

Considering the formula for the nucleation rate per unit volume due to autocatalytic nucleation ${\left(\frac{\mathrm{dN}}{\mathrm{dt}}\right)}_{A}$ proposed by Ravi, Sietsma and Santofimia [57]:

(4) $${\left(\frac{\mathrm{dN}}{\mathrm{dt}}\right)}_{A}=\frac{\mathrm{kT}}{h}N_{\mathrm{tA}}\exp\left(-\frac{Q_{A}^{*}}{\mathrm{kT}}\right)$$

where k is the Boltzmann’s constant, h is the Planck’s constant, $N_{\mathrm{tA}}$ is the number density of potential autocatalytic nucleations sites at given time t, $Q_{A}^{*}\;$is the activation energy for autocatalytic nucleation, and T is the isothermal transformation temperature, we see that the changes in the autocatalytic stage of the bainitic transformation were caused by the $N_{\mathrm{tA}}$ and $Q_{A}^{*}$ changes.

Changes in $N_{\mathrm{tA}}$ were associated with changes in the differences in the kinetics of the bainitic ferrite nucleation on PAGBs. This is described in an earlier chapter. The changes in $Q_{A}^{*}$ were also caused by changes in the kinetics of bainitic ferrite nucleation on PAGBs. They consisted of a change in the distribution of forces between competing phenomena, autocatalytic nucleation of bainitic ferrite on ferrite–austenite interfaces, and the accompanying redistribution in the austenite matrix of extruded carbon atoms.

When analysing the influence of IAT on bainitic transformation in high carbon steel, Ravi et al. did not notice the acceleration of bainitic transformation at the stage of autocatalytic growth. Moreover, their research shows that the final amount of bainitic ferrite was similar for all considered variants [22]. In the case of low-carbon steel, they noticed that the increase in time and the decrease in IAT temperature led to an increase in the rate of transformation acceleration in the autocatalytic stage of growth [23]. The decrease in the IAT temperature had the opposite effect—Figure 4 and Figure 5. They did not signal a change in the final amount of bainitic ferrite caused by the use of IAT, nor the refinement of the final microstructure due to IAT that we noticed. These differences testify to the complexity of the impact of IAT on the subsequent bainitic transformation as well as the impact effect of steel’s chemical composition on changes occurring in the material during IAT.

## 5. Conclusions

The subject of this work was to determine the influence of intermediate annealing treatment (IAT) on the kinetics of a subsequent bainitic transformation in the X37CrMoV5-1 steel. It was noticed that IAT use affected the incubation time and the kinetics of the autocatalytic stage of the bainitic transformation. In order to quantitatively analyse these changes, a method was proposed that consisted of fitting the Bolzann sigmoids to dilatometric curves corresponding to the bainitising process. The representation of these curves using the dt and t_0.5_ parameters brings information about the transformation kinetics. Studies have shown a strong influence of the IAT temperature and time on the direction and strength of changes in the kinetics of bainitic transformation. For variants with IAT at temperatures 800, 700, and 600 °C, a shortening of the incubation time of bainitic transformation and an acceleration at the stage of autocatalytic growth was observed. The longer the IAT segment was, the stronger these processes occurred. In the case of IAT at 500 °C, the opposite effect was observed, i.e., the prolongation of the bainitic transformation incubation time and the deceleration of the autocatalytic growth stage compared to the variant without IAT. These effects were stronger with a longer IAT. The LM and SEM microscopic observations, EDS analysis, and computer simulations indicated that during the IAT, M_23_C_6_ type carbides were precipitated. The presence of these carbides causes changes in the kinetics of bainitic. By appropriately selecting the temperature and time of the IAT, it is possible to shape the distribution, concentration, and morphology of the precipitations, and therefore to control the bainitic transformation kinetics. IAT that is properly carried out can lead to a significant acceleration of the bainitic transformation, a higher final rate of transformation, and a highly refined microstructure. Furthermore, as shown by the TEM observations, it is possible to obtain nanobainite using IAT. From an industrial perspective, this is very beneficial because it could shorten the heat treatment processes and obtain favourable mechanical properties. The influence of IAT on the kinetics of bainitic transformation is a relatively new issue. It requires further research, especially in the context of the impact of such heat treatments on the mechanical properties of bainitic steels.

## Figures and Tables

**Table 1:** Chemical compositions of EN X37CrMoV5-1 steel (in wt %).

C	Si	Cr	Mn	Mo	Ni	V
0.37	1.16	4.95	0.43	1.22	0.26	0.40

**Table 2:** IAT parameters of the performed treatments and the treatment designations.

Treatment	IAT Temperature and Time	Designation
quenching	800 °C, 240 min	M-800-240
	800 °C, 30 min	M-800-30
	700 °C, 240 min	M-700-240
	700 °C, 30 min	M-700-30
	600 °C, 240 min	M-600-240
	600 °C, 30 min	M-600-30
	500 °C, 240 min	M-500-240
	500 °C, 30 min	M-500-30
	no IAT	M-no-IAT
austempering	800 °C, 240 min	B-800-240
	800 °C, 120 min	B-800-120
	800 °C, 30 min	B-800-30
	700 °C, 30 min	B-700-30
	700 °C, 15 min 40 s	B-700-15
	600 °C, 240 min	B-600-240
	600 °C, 30 min	B-600-30

**Table 3:** Summary of Bolztmann sigmoid fitting parameters of dilatometric bainitising curves.

IAT Variant	dt [s]	t_0.5_ [s]	Adj. R^2^
B-800-240	626.1 ± 4.6	12.7 ± 21.4	0.99694
B-800-120	636.2 ± 1.6	934.7 ± 3.7	0.99822
B-800-30	715.7 ± 2.8	2120.2 ± 3.9	0.99864
B-700-30	973.1 ± 3.2	1386.9 ± 7.1	0.99947
B-700-15	945.0 ± 1.8	1691.0 ± 3.4	0.99895
B-600-240	917.9 ± 3.1	3623.0 ± 3.6	0.99932
B-600-30	930.1 ± 1.4	3637.1 ± 1.6	0.99986
B-500-240	1190.1 ± 1.5	4930.4 ± 1.5	0.99987
B-500-30	1109.9 ± 2.3	4690.0 ± 2.4	0.99975
B-no-IAT	1170.0 ± 1.3	3957.2 ± 1.5	0.99987

## Data Availability

All data included in this study are available upon request by contact with the corresponding author.
